# Electrical impedance myography combined with quantitative assessment techniques in paretic muscle of stroke survivors: Insights and challenges

**DOI:** 10.3389/fnagi.2023.1130230

**Published:** 2023-03-16

**Authors:** Ze Gong, Wai Leung Ambrose Lo, Ruoli Wang, Le Li

**Affiliations:** ^1^Research & Development Institute of Northwestern Polytechnical University in Shenzhen, Shenzhen, China; ^2^Institute of Medical Research, Northwestern Polytechnical University, Xi’an, China; ^3^Department of Rehabilitation Medicine, First Affiliated Hospital of Sun Yat-sen University, Guangzhou, China; ^4^KTH MoveAbility Lab, Department of Engineering Mechanics, KTH Royal Institute of Technology, Stockholm, Sweden

**Keywords:** electrical impedance myography, stroke, skeletal muscle, spasticity, ultrasonography

## Abstract

Aging is a non-modifiable risk factor for stroke and the global burden of stroke is continuing to increase due to the aging society. Muscle dysfunction, common sequela of stroke, has long been of research interests. Therefore, how to accurately assess muscle function is particularly important. Electrical impedance myography (EIM) has proven to be feasible to assess muscle impairment in patients with stroke in terms of micro structures, such as muscle membrane integrity, extracellular and intracellular fluids. However, EIM alone is not sufficient to assess muscle function comprehensively given the complex contributors to paretic muscle after an insult. This article discusses the potential to combine EIM and other common quantitative methods as ways to improve the assessment of muscle function in stroke survivors. Clinically, these combined assessments provide not only a distinct advantage for greater accuracy of muscle assessment through cross-validation, but also the physiological explanation on muscle dysfunction at the micro level. Different combinations of assessments are discussed with insights for different purposes. The assessments of morphological, mechanical and contractile properties combined with EIM are focused since changes in muscle structures, tone and strength directly reflect the muscle function of stroke survivors. With advances in computational technology, finite element model and machine learning model that incorporate multi-modal evaluation parameters to enable the establishment of predictive or diagnostic model will be the next step forward to assess muscle function for individual with stroke.

## Introduction

Population aging is an urgent global issue, accompanied with a substantial burden for age-related diseases. It was reported that about 1,000 among every hundred thousand people aged 70 years or older suffer stroke in the world (Feigin et al., 2021). As one of the leading causes of death and disability in the elderly, stroke has long been of interest, particularly in motor function recovery (Lo, 2014; Feigin et al., 2022). Approximately 80% of stroke survivors suffer from motor dysfunction, including hemiparesis, incoordination, and spasticity, which severely hinder their daily life (Lora-Millan et al., 2022). How to improve motor control and movement quality are challenging clinicians and researchers (Patten et al., 2004). The smooth control of movement relies not only on the integrity of neural pathways, but also on the function of the musculoskeletal system. Therefore, assessing muscle function is essential to understand factors underlying motor dysfunction after stroke. Typically, muscle function is affected by its structural changes (Power et al., 2013). For stroke survivors, disrupted synaptic inputs to the motor neurons commonly resulted in muscle fiber loss, intramuscular fat infiltration and connective tissue growth (Scherbakov et al., 2013; Li et al., 2016d). All of these changes could influence the intrinsic muscle properties (Li et al., 2017c). In turn, the evaluation of muscle properties may reflect the muscle function and improve current knowledge on the mechanisms of muscle dysfunction in stroke survivors.

Electrical impedance myography (EIM), a non-invasive evaluation instrument, aims at quantifying the intrinsic passive electrical properties of muscle (Rutkove, 2009). Alterations in the voltage caused by changes of diseased muscle conductivity and permittivity are the basis of EIM (Foster and Schwan, 1989). It has been studied that EIM parameters, resistance, reactance and phase angle, are able to reflect specific physiological information on the composition and micro structures of muscle tissue respectively, e.g., myofiber membrane, extracellular and intracellular fluids (Castizo-Olier et al., 2018; Fu and Freeborn, 2020). In clinical setting, electrical impedance variables have been verified as reliable biomarkers for neuromuscular diseases, such as amyotrophic lateral sclerosis and muscular dystrophy (Rutkove and Sanchez, 2019). Clark et al. (2021) reviewed the feasibility of EIM in age-related diseases and affirmed its potential clinical value in the assessment of muscle health concerning aging. In elderly with stroke, we reported significant changes in the passive electrical behavior of muscle (Li et al., 2017c). Similar results were also observed in patients with spinal cord injury (Li et al., 2017a,b). How to interpret the changes recorded by EIM has always been the focus of clinical application. With the help of animal experiments, electrical impedance data have been proved to be involved with the structural degeneration of muscle, especially in the number of muscle fibers, state of the cell membranes and the proportion of fat and connective tissue infiltration (Pandeya et al., 2021b,a). From a functional perspective, Li et al. (2016a) observed a significant relationship between EIM parameters and force production in mice. It follows that EIM has the potential to establish the relationship between muscle function and microstructural properties, providing physiological evidence for muscle evaluation. Furthermore, our team also demonstrated excellent sensitivity of EIM technique in the measurement of alterations on muscle composition in stroke patients (Li et al., 2017d). Coupled with excellent robustness and convenience, EIM holds a high potential for clinical assessment of muscle function (Martinez-Gonzalez et al., 2020; Rutkove et al., 2021). However, there have been various contributors to muscle dysfunction in persons after stroke and the passive electrical property measured by EIM may not fully reflect the paretic muscle changes.

Given that the factors leading to muscle dysfunction are uncertain and the sensitivity of techniques varies at different pathological features of muscle, there is a need for combined assessments of muscle function in clinical settings. Apart from muscle properties itself, its dysfunction tends to initially derive from the denervation of muscle after stroke (Scherbakov et al., 2013) and follows that nerve-related factors are indispensable for muscle function evaluation. Thus, EIM alone is not sufficient for assessment of muscle function comprehensively. Additionally, the clinical value of EIM remains controversial (Rutkove et al., 2021; Sanchez et al., 2021). Sanchez et al. (2021) pointed out that EIM measurements is likely to be influenced by the accuracy of measurement localization and the specificity of the target tissue (i.e., subcutaneous fat tissues and skin tissues affect the measurements). Although previous work has drawn links between EIM values and electrophysiological, histological, imaging and functional outcomes (Rutkove et al., 2014a; Hamel et al., 2020), the interpretation of EIM values is suggested to be limited and EIM cannot substitute for other assessments in many cases. It is noted that EIM is only capable of assessing superficial muscles but not the deep muscle layer, owing to the limited penetrative ability of electrical current (Hu et al., 2021c). Therefore, the EIM findings need to be interpreted with caution in clinical decision-making.

**FIGURE 1:**
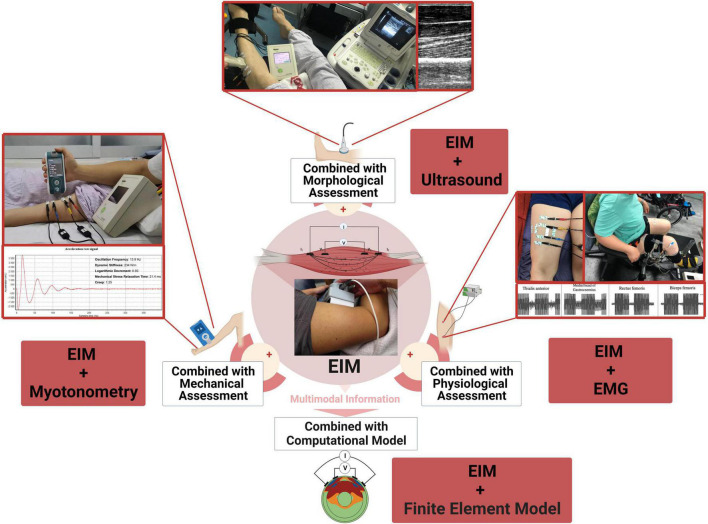
Conceptual illustration of combining EIM with other technologies for muscle function evaluation.

Over the past decades, quantitative assessment of muscle function gained popularity due to their precision and objectivity. These assessment methods evaluate various aspects influencing muscle function, including morphological, mechanical and contractile properties. Here, we discuss the potential utility for combined assessments of EIM with other quantitative techniques and provide the insights and challenges for evaluating muscle function in patients with stroke (Figure 1). The main findings are displayed in the Table 1.

**TABLE 1:** The main findings of each section.

Combining EIM with	Assessment	Study	Main findings
Morphological Property Assessment	Ultrasound	Rutkove et al., 2010; Rutkove and Darras, 2013; Johnson et al., 2018; Hu et al., 2019; Longo et al., 2021	• Clinical measurements from EIM and ultrasound were verified mutually. • Both methods could be applied applied to depict muscle architecture and improve disease diagnosis accuracy.
		Sung et al., 2013; Li et al., 2016b	• The application of ultrasound could guide the utilization of EIM for improved accuracy and stability. • The application of ultrasound could evaluate the influence of subcutaneous fat layer thickness on EIM measurements.
		Rutkove et al., 2014b; Shklyar et al., 2015; Hobson-Webb et al., 2018, 2021; Roy et al., 2019	• Significant associations were observed between measurements obtained from EIM and those obtained from quantitative ultrasound. • Combining the data from these two techniques has been shown to yield more accurate muscle function measurements.
	MRI	Hamel et al., 2020; Albano et al., 2022a,b	• Establishing the correlation of the EIM with structural MRI features. • Comparing EIM and MRI measurements in assessing muscle composition.
	CT	Anderson et al., 2014	• Paraspinal electrical impedance myography phase was positively correlated with paraspinal attenuation based on CT, which established the relationship between EIM and CT.
	DXA	McLester et al., 2018; van Rassel et al., 2019; Czeck et al., 2020	• EIM has been proved to be a relatively reliable method to estimate body fat percent compared to DXA.
Mechanical Property Assessment	NeuroFlexor	Leng et al., 2021	• Neural component was found significantly associated with reactance recorded by EIM. • Viscosity component was significantly associated with phase angle.
	Myotonometer	Hu et al., 2021b	• The creep and relaxation time obtained from myotonometer was significantly associated with reactance recorded by EIM.
Contractile Property Assessment	Strength test	Rutkove et al., 2014a; Shellikeri et al., 2015; Zaidman et al., 2015	• The observed relationship between EIM and strength tests suggested that EIM had the potential as a meaningful measure in the diagnosis of neuromuscular diseases.
		Li et al., 2016a,c; Coutinho et al., 2020	• The combination of EIM and strength test has the potential to directly reveal the changes in the force generation properties of the target muscle.
	EMG	Li et al., 2013, 2014, 2017f; Arnold et al., 2016, 2017; de Carvalho and Swash, 2016; Kolb et al., 2016; Zong et al., 2018	• EIM was closely associated with various electrophysiological measures, especially compound muscle action potential from EMG. • EIM is a promising biomarker in assessment of muscle function in neuromuscular diseases and its relationships with other electrophysiological measures have been well-established in various neuromuscular diseases.
		Hu et al., 2021a; Ngo et al., 2022	• Applying EIM and EMG simultaneously could provide more comprehensive information of the inherent properties and muscle activation alteration.
Computational Model	Finite element model	Ahad and Rutkove, 2009; Wang et al., 2011; Jafarpoor et al., 2011; Pacheck et al., 2016	• Finite element models have been demonstrated as a capable method to investigate the biophysical mechanisms of EIM in various diseases.
		Jafarpoor et al., 2013; Baidya and Ahad, 2016; Rutkove et al., 2017; de Cardoner et al., 2021; Schooling et al., 2020; Luo et al., 2022; Schrunder et al., 2022	• Finite element analysis provides a basic methodology to optimize electrode configuration for EIM
	Machine learning model	Srivastava et al., 2012; Pandeya et al., 2022	• Combining biomarkers from EIM or other assessments could enhance the diagnostic performance of machine learning models.
		Kapur et al., 2018a,b; Pandeya et al., 2021a,b; Cheng et al., 2022	• Combining EIM and other biomarkers through machine learning can improve diagnostic accuracy for estimating muscle function parameters.

## Combination of EIM and morphological assessment

The loss of muscle function is typically accompanied with changes of muscle morphology after stroke (Metoki et al., 2003). Common morphological features found in patients with stroke include a reduction of muscle cross-sectional area, mass, volume, pennation angle and fascicle length. Sions et al. (2012) proposed that the decrease in the muscle cross-sectional area would eventually lead to the loss of muscle strength. Moreover, intramuscular fat is also found associated with greater muscle torque (Goodpaster et al., 2001). It is possible that muscle morphological changes reflect its dysfunction indirectly. Assessments of muscle morphology adopted in clinical practice involve magnetic resonance imaging (MRI), computed tomography (CT), dual-energy x-ray absorptiometry (DXA) and ultrasound imaging. Although these tools could provide reliable and accurate information on the morphology (Heymsfield et al., 2014; Clark et al., 2018), their clinical utility is limited by complex operation procedure, data analysis and expenses (Bazzocchi et al., 2016; Tavoian et al., 2019; Clark et al., 2021). Further, these are also challenges to incorporate with other kinetic instruments such as torque dynamometers simultaneously. Morphological measurement alone does not give the overall muscle status qualification.

Faced with above limitations, increasing number of researchers turned their sights on EIM to search for alternative verifiable biomarkers of muscle status. There is evidence that the application of EIM can estimate well for the muscle cross-sectional area (Pandeya et al., 2021a), myofiber size (Arnold et al., 2017) and arrangement of myofibers (Garmirian et al., 2009). In subacute stroke survivors, we have demonstrated significant associations of electrical impedance data with ultrasonography parameters (Hu et al., 2019). Muscle fascicle length, thickness and pennation angle recorded by ultrasound have been recognized to effectively illustrate the muscle status in poststroke impairments (Li et al., 2007). According to previous reports, the EIM and ultrasound could be applied jointly to depict muscle architecture (Rutkove et al., 2010; Rutkove and Darras, 2013; Johnson et al., 2018; Hu et al., 2019; Longo et al., 2021). With the development of quantitative ultrasound, its relationship with EIM is playing an increasing important role in improving disease diagnosis (Rutkove et al., 2014b; Shklyar et al., 2015; Hobson-Webb et al., 2018, 2021; Roy et al., 2019). Shklyar et al. (2015) reported that composite data from EIM and quantitative ultrasound can better predict the muscle function compared with individual measurement data. Furthermore, several studies have established the relationship between EIM variables and structural features of muscles, as measured by MRI, CT, and DXA. Those studies also further supported the potential value of EIM in muscle structure assessment (Anderson et al., 2014; McLester et al., 2018; van Rassel et al., 2019; Czeck et al., 2020; Hamel et al., 2020; Albano et al., 2022b,a). While aforementioned correlation was validated, not all findings are fully consistent, since different techniques are sensitive to different pathological features of muscle (Roy et al., 2019). There is a need for combined assessment to assist in clinical muscle evaluation or diagnosis. The correspondence of variables between EIM and other quantitative techniques provide a foundation for cross-validation in muscle assessment and facilitates the accurate evaluation of muscle function.

In addition to accuracy, combined assessments could improve the understanding of mechanisms of muscle dysfunction. Parameters from EIM, including resistance, reactance and phase angle, are reported to be closely associated with muscle’s components, extra- and intra-cellular fluids, tissue interfaces and the integrity of cell membrane (Esper et al., 2006). EIM data could be an important compliment to the morphological assessment. It is these relationships that help clinicians to understand the physiological significance behind morphological variables, further driving the development of precision therapy.

The combination with other assessment methods would advance the application of EIM. As the electrical impedance data is susceptible to subcutaneous fat tissue, our team investigate the effect of subcutaneous fat on EIM with ultrasound assistance and found appropriate electrode configuration to reduce the impact (Li et al., 2016b). Sung et al. (2013) found that the reactance, one of the EIM parameters, was least affected by subcutaneous fat when the EIM was applied with the aid of ultrasound. Conventional muscle morphological assessment may guide the application of EIM to improve accuracy and stability. If clinicians performed conventional assessments (e.g., ultrasound) and identify the amount of subcutaneous fat thickness in advance, they would select the appropriate electrode configuration to improve the accuracy and stability of measurement.

## Combination of EIM and mechanical property assessment

Dystonia is a common muscle dysfunction for stroke patients, characterized by reduced or increased muscle tension (Tsai et al., 2020). These deficits could be attributed to inherent properties of contractile elements, viscoelastic properties of musculotendinous units and abnormalities of reflex excitability (Chuang et al., 2012). Thus, the assessment of both neural and mechanical properties is critical to provide a full picture of muscle function. Muscle strength and joint range of motion are often adopted as indirect measurements of muscle mechanical properties (Brandenburg et al., 2014). However, these indirect methods can only evaluate mechanical properties across the joint instead of individual muscles, potentially limiting precision assessment.

Myotonometry was proposed as a way to quantify individual muscle mechanical properties including stiffness, elasticity, and muscle tone (Chuang et al., 2013). The principle behind myotonometry is to apply brief impulses over the muscles to induce oscillations, which are recorded to calculate the parameters of tone, creep, stiffness and decrement (Lo et al., 2017). There have been several studies which reported the reliability and sensitivity of myotonometric measurement in stroke rehabilitation (Chuang et al., 2012; Li et al., 2017e). Previous work highlighted the potential benefit of applying myotonometry to quantify muscle mechanical properties. However, there is uncertainty regarding the interpretation of myotonometric data since there is little information regarding the normative values that could be used for reference. There are also uncertainties in the physiological contributing factors to alteration of muscle mechanical properties (Lo and Li, 2020). Thus, myotonometry is not recommended to be applied alone. Establishing the relationship between physiological structures and muscle mechanical properties remains necessary for researchers or clinicians to optimize therapeutic regimens. Unfortunately, it is not realistic to widely use muscle biopsy for assessing muscle structures in the clinic. According to our previous studies, reactance recorded by EIM was significantly associated with the creep and relaxation time from myotonometer in patients who suffered cervical spinal cord injury (Hu et al., 2021b). The former was reported to be related to myocyte atrophy (Li et al., 2012), which is also likely to lead to the reduction of muscle contractile ability. This change often reflects as higher relaxation time and creep in myotonometry. Similar relationships can also be observed in patients with stroke, where muscle viscosity was significantly associated with phase angle (Leng et al., 2021). These findings offer us an opportunity to interpret the mechanical property alterations from a physiological structural point. Whether or not the combined assessments may improve assessment of muscle function warrant further investigation.

Spastic hypertonia stems from the neural and non-neural factors is frequently observed in stroke survivors (Luo et al., 2020). Conventionally, the neural components are defined as the intrinsic excitability of motoneurons themselves, while non-neural components can be represented as the resistance caused by inertia, elasticity, and viscosity of the moved body part (Lindberg et al., 2011). Distinguishing neural and non-neural components is critically important for developing targeted intervention regimens. Spastic muscle mainly caused by neural contribution is suited to treatment against nerve-pathway (e.g., nerve block) (Bovend’Eerdt et al., 2008). While varieties of approaches proposed to distinguish the neural factors (Lee, 2002; Kamper et al., 2003; Musampa et al., 2007; Alibiglou et al., 2008), it is hard for these methods to truly distinguish neural components and their clinical utilities are limited by operational complexity (Wood et al., 2005). Lindberg et al. (2011) built a biomechanical model, Neuroflexor model, to quantify the neural and non-neural contributions by measuring the resisting force during passive extension at slow and fast speeds. Our previous studies reported the higher neural, elastic and viscosity components of the Neuroflexor method on the affected side compared to non-affected side in stroke patients (Leng et al., 2021). Combined with EIM, we found EIM parameters of reactance was inversely proportional to neural component, while phase angle was proportional to viscosity component in spastic muscle, potentially supporting the feasibility of combined assessment based on EIM and Neuroflexor in stroke patients. This relationship also provides the basis to explore the effect of nervous factors on peripheral muscular structures.

## Combination of EIM and contractile property assessment

Motor function deficits, those caused by muscle weakness, can only be observed during voluntary muscle contraction. Thus, understanding of muscle contractile property (i.e., shortening velocity and maximal force) is of great interest (Gao et al., 2005). Active movement tests, such as the handgrip test, are easily performed but their results are susceptible to be affected by the integrity of the nervous system and subjective factors (Carson, 2018). That is, these tests fail to accurately distinguish the true cause of muscle dysfunction (nerve or muscle related). Prior studies have found that muscle weakness was associated with the loss of muscle fiber and increase in intramuscular adipose tissue, caused by muscle denervation, disuse, and atrophy (Scherbakov et al., 2015; Berenpas et al., 2017). Numerous researches have reported the association of muscle strength with various macrostructures clinically (Petterson et al., 2008; Kellis et al., 2021). However, these studies cannot explore muscle dysfunction at a micro level. Building on strength testing, the addition of EIM, which reflects indirectly the properties of cell membranes and the content of extracellular and intracellular water (Faes et al., 1999; Shiffman and Rutkove, 2013), probably provide advantages for the exploration of mechanism behind muscle dysfunction. Several studies have explored the relationship between the EIM and strength tests in neuromuscular diseases, which suggested that EIM may serve as a valuable measure for diagnosing neuromuscular conditions (Rutkove et al., 2014a; Shellikeri et al., 2015; Zaidman et al., 2015). Our findings indicated that this combination could also reveal the changes in properties of target muscle directly during force generation (Li et al., 2016c). Likewise, the relationship between the EIM data and force production was verified in animal experiments (Li et al., 2016a; Coutinho et al., 2020). These insights might be beneficial when further extended to clinical assessments of alterations in force production after stroke.

Actually, the generation of force is inseparable from active muscle contraction, depending on the number and size of active motor units, as well as the rate and timing of their discharge (Herzog et al., 2015; Duchateau and Enoka, 2016). In the past decades, EMG assessment has been recognized as a gold standard for evaluating the recruitment and activation patterns of muscle (Schuermans et al., 2014). Compared with strength testing, EMG records electrophysiological data to explain the potential reasons for the impairment of muscle contractile function. However, EMG is limited to the evaluation of active electrophysiological property without concerning passive property. From a mechanistic perspective, the relationships between EIM data and EMG parameters, especially compound muscle action potentials, have been demonstrated in persons with various neuromuscular disease over the years (Li et al., 2013, 2014, 2017f; Arnold et al., 2016, 2017; de Carvalho and Swash, 2016; Kolb et al., 2016; Zong et al., 2018). Our team has demonstrated the potential of the EIM and EMG to jointly evaluate lower-extremity muscle function and to assess the impact of rehabilitation training in a clinical setting (Hu et al., 2021a; Li et al., 2022). Both active and passive muscle properties were assessed at the same time, which could advance the understanding of muscle dysfunction. Ngo et al. (2022) proposed a novel device that can combine EIM and EMG measurements simultaneously, offering a more robust method for muscle status assessment even in the presence of artifacts.

## Combination of EIM and computational model

Standards for EIM parameters in clinical applications are still lacking, especially when the comparison of data across individuals is considered. As this technique is sensitive to muscle morphological, electrical properties and the electrode configuration (Schrunder et al., 2022), it is then necessary to identify the impact of these factors and to explore approaches to minimize their effects. Computational modeling that incorporates detailed finite element models has been proven capable in studying the biophysical mechanisms of EIM and optimize the design of electrode arrangements (Ahad and Rutkove, 2009; Jafarpoor et al., 2011, 2013; Wang et al., 2011; Baidya and Ahad, 2016; Pacheck et al., 2016; Rutkove et al., 2017; Schooling et al., 2020; de Cardoner et al., 2021; Luo et al., 2022; Schrunder et al., 2022). Wang et al. (2011) utilized a finite element model to investigate the correlation between changes in surface impedance and electrical properties of the muscle, offering valuable insights into the biophysical mechanisms underlying the EIM. Schrunder et al. (2022) simulated muscle tissues and impacts from anthropometric variations and EIM electrode placements. Baidya and Ahad (2016) applied a finite element model to determine an optimized EIM electrode configuration which could balance both stability and sensitivity. However, previous finite element studies seldom focus on the application of EIM in stroke survivors. This work needs to be studied based on the actual data from individuals with stroke in the future.

As the data of muscle assessment becomes increasingly diverse, how to identify and integrate valuable information are attracting more and more attention. Multimodal learning has grown rapidly with the continuous progress of multimodal information input and algorithm (Baltrusaitis et al., 2019). Drawing on a muscle perspective, the multi-modality fusion of different assessments is considered to substantially drive the clinical assessment advancement with the help of machine learning. Srivastava et al. (2012) refined biomarkers from EIM and quantitative muscle ultrasound on the basis of machine learning to successfully classify the muscle affected by spinal muscular atrophy. Pandeya et al. (2022) demonstrated that using multifrequency EIM values instead of single-frequency values can improve classification performance of machine learning. Similarly, Cheng et al. (2022) reported that machine learning could estimate the total mass of muscles with the EIM and anthropometric parameters. The use of machine learning approaches has allowed for the prediction of myofiber size, cross-sectional area, and connective tissue deposition using the EIM measurement (Kapur et al., 2018b,a; Pandeya et al., 2021b,a; Cheng et al., 2022). Addressing different requirements, specific quantitative assessment parameters are selected to be combined with electrical impedance data. Currently, the combined assessments based on machine learning are rarely employed to detect muscle changes in stroke patients. Establishing a prediction or diagnostic model based on machine learning to assess muscle function for patients with stroke will be the next step forward.

## Conclusions and future directions

Overall, the application of combined assessments based on EIM with quantitative techniques is a promising direction to assess muscle function in stroke patients. In view of different purposes, distinct combinations are selected to evaluate specific aspect of muscle function. Combining different techniques could improve the accuracy and comprehensiveness of muscle assessment, and also advance the understanding of physiological mechanism that underpins muscle dysfunction.

Despite the potential of combined assessments in clinical application, several challenges remain at both the mechanistic and clinical levels.

At the mechanistic level, there were lacks of robustness in identifying the relationship between EIM and standard parameters of other assessments, thereby hindering the application of EIM in clinical settings. Establishing this relationship requires a deeper understanding of the biophysical mechanisms of EIM, which requires further investigation. Secondly, most studies cannot provide a causal interpretation, and the sequence of alterations in variables remains unclear. To address this issue, more longitudinal studies with longer period of follow-ups are required. Thirdly, research efforts in the field of muscle function assessment have been uneven, with the combination of EIM and mechanical property assessment receiving less attention and requiring further exploration (see Table 1). Furthermore, enabling clinical application also raises unique challenges. On the one hand, selecting effective combination of assessments for a specific purpose remains nontrivial for clinicians and researchers. As sensitivity varies across different assessments, selecting appropriate combination and extract essential features from multiple types of data are particularly crucial. The advancement of various computational techniques can assist in identifying, extracting, and analyzing multimodal information of muscle function, leading to the standardization in the clinical practice. On the other hand, the estimation accuracy of EIM has been a concern for years. Good accuracy relies on proper electrode configuration where finite element modeling could inform clinicians in an appropriate electrode configuration and placement. However, performing modeling for each patient based on individual MRI or CT scans is impractical. Therefore, building prediction models based on multimodal information is necessary. In summary, both opportunities and challenges exist in mechanistic studies and clinical applications for EIM.

## Author contributions

LL and ZG: conceptualization. ZG, WL, RW, and LL: methodology. ZG and WL: writing – original draft preparation. WL, RW, and LL: writing – review and editing. All authors contributed to the article and approved the submitted version.
