# Supplementary material for: Electrical impedance myography combined with quantitative assessment techniques in paretic muscle of stroke survivors: Insights and challenges
Source: Front Aging Neurosci. 2023 Mar 16;15:1130230. doi: 10.3389/fnagi.2023.1130230 (PMC10069712; doi:10.3389/fnagi.2023.1130230)
Supplement: Supplementary file 1 [file Data_Sheet_1.PDF]

Supplemental Figure 1 (S-Fig.1)

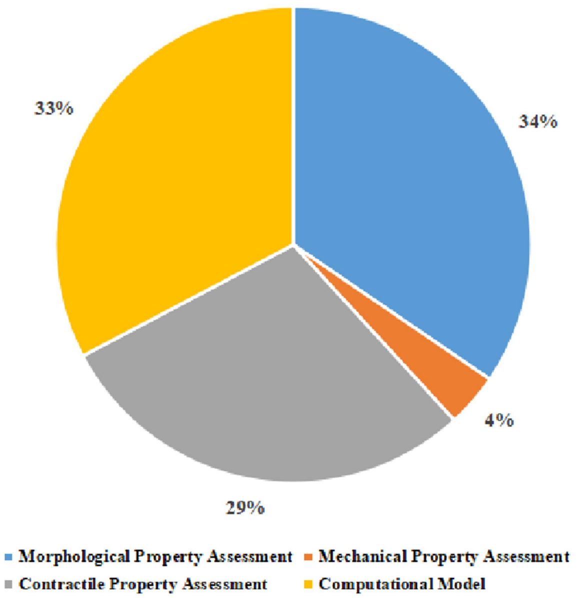

Supplemental Figure 1. The distribution of publications in each type of combination.
